# Welding, Joining, and Additive Manufacturing: Experiments, Materials, and Modeling

**DOI:** 10.3390/ma18020445

**Published:** 2025-01-19

**Authors:** Swarup Bag, C. P. Paul

**Affiliations:** 1 Department of Mechanical Engineering, IIT Guwahati, Guwahati 781039, Assam, India; 2 Raja Ramanna Centre for Advanced Technology, Indore 452013, Madhya Pradesh, India; paulcp@rrcat.gov.in

## 1. Introduction

With the advancement of technologies, welding and joining have become an integral part of advanced manufacturing systems. The joining of new materials, dissimilar materials, composite materials, different geometric configurations, and miniature components poses significant challenges to the established joining technologies. The industry’s primary concern is establishing sustainable joining technologies for non-conventional forms of materials and using these energy sources for developing additive or printing technologies through experiments and mathematical modeling. Although joining metallic materials through deposition is an established process, strategic material transport becomes indispensable to establish a layer-by-layer manufacturing process. Material deposition from the principle of fusion welding in a controlled manner is the backbone for wire arc additive manufacturing (WAAM) technology. However, mathematical modeling of welding and additive manufacturing processes helps to establish the in situ physical behavior of the process. In contrast, simple experiments do not always allow for the reaching of any definite conclusion. It requires an understanding of the phenomenological behavior of thermo-fluid or thermo-mechanical analysis with the application of material science. In addition, the establishment of structure–property relation requires proper characterization techniques of the components, as well as measurement of temperature. Given the anticipated objective, this Special Issue presents several articles in the diverse directions of modeling, characterization, and measurement techniques of welding, joining, and additive manufacturing issues for materials ranging from simplistic to exotic.

## 2. Short Description of the Articles Presented in This Special Issue

The research on welding, joining, and additive manufacturing processes builds up through the coverage of ten individual articles broadly focused on experiments and physics-based models of very critical aspects. This Special Issue mainly focuses on fusion welding and wire arc additive manufacturing (WAAM). The numerical solution of the process starts with the governing equations and boundary conditions using suitable solution techniques. The analytical solution for process stability of fusion welding and additive manufacturing is the most promising input of the present editorial. In addition, the bubble collapsing of fusion welding is invariably an important feature. The heat source model of fusion welding at cryogenic conditions brings new dimensionality over the conventional model. The temperature measurement technique for the additive manufacturing process is an excellent contribution to advancing the current manufacturing system. The mechanical and microstructural characterization of welded and additively manufactured components of advanced materials brings innovative information to the manufacturing community. All these papers have been peer-reviewed for publication in this Special Issue. A short review of all these papers is presented as follows. The first six papers are relevant to welding and joining, and the remaining four papers describe the critical aspects of additive manufacturing.

The paper entitled “Comparative study on the behavior of keyhole in analogy welding and real deep penetration laser welding” depicts the experimental simulation of keyhole formation to understand the mechanism [1]. The argon gas stream is passed through different liquid mediums like water, NaCl solution, and Ga–In–Sn alloy to produce the keyhole, and the analysis is performed from the images of a high-speed camera. The compressive pressure consists of gas jet-induced pressure, surface tension at the interface, and hydrostatic pressure at the liquid surface, which is compared to the vapor pressure of the actual deep penetration laser welding system. The liquid pressure in analogy welding and vapor pressure by evaporation in laser welding are the main driving forces for opening the keyhole, whereas the hydrostatic pressure and surface tension promote the closing of the keyhole.

The paper entitled “Analytical simulation of the microbubble collapsing in a welding fusion pool” shows the ultrasound treatment in the weld pool to influence the solidification behavior and refine the microstructure in a vibration-assisted welding process [2]. The approach lies in finding the optimum position of the working plate to remotely vibrate it such that the ultrasound system will create a cavitation vibration at the weld pool. It is concluded that 20 kHz frequency over a 0.1 s solidifying phase is sufficient time to make the microbubbles burst in the semifluid, which contributes to grain refinement and better shaping of intermetallic phases.

The paper entitled “An improved method for deriving the heat source model for FCAW of 9% Nickel steel for cryogenic tanks” describes the integrated evolutionary optimization algorithm with finite element-based heat transfer analysis [3]. The joining method is adopted here as flux-cored arc welding (FCAW). The temperature is experimentally measured at a series of thermocouple points and is compared with computed data to minimize the error. This inverse analysis is directed toward finding the most suitable double-ellipsoidal heat source parameters. It is concluded that the adjustment of the heat source parameters is befitted with a relatively coarse mesh of reduced computational time. This simplified heat conduction model accounts for a large set of heat source parameters to reach an optimum level with a low computational time.

The paper entitled “Microstructure and mechanical properties of steel and Ni-based superalloy joints for rotors of high-speed electric motors” portrays the mechanical and microstructural characterization of dissimilar joints of magnetic (stainless steel) and non-magnetic (Inconel) laminated sheets [4]. This welding method is applicable for high-speed electric motors where laminated sheets are joined by vacuum braze and hot isostatic pressing (HIP) processes. The results show that both methods are equally efficient. The finite element simulation of stress distribution is performed at the operating speed of the rotor. The minimum shear strength of the joint is estimated, which is close to the experimental results, essentially showing the effectiveness of the developed numerical model.

The paper entitled “Microstructural and performance analysis of TP304H/T22 dissimilar steel welded joints” defines the effectiveness of dissimilar austenitic stainless steel and austenitic/ferritic stainless-steel pipes for high-temperature (550 °C) applications [5]. The weak position of the welded joints is located through thermo-mechanical simulation of tensile specimens. A defect-free environment, devoid of any creep cavities and intergranular cracks, is achieved in the present work. It is concluded that the fracture occurs either in the base material or in the fusion zone of the austenitic stainless steel side at a high-temperature tensile load.

The paper “Assessment of changes in abrasive wear resistance of a welded joint of low-alloy martensitic steel using microabrasion test” illustrates the abrasion wear resistance of a welded joint of low-alloy martensitic steel [6]. The samples are prepared by multipass MAG (metal-active gas) welding with a preheat temperature of 50 °C and interpass cooling to 225 °C. The wear test is carried out by the ball-cratering method using abrasive slurry over the weld cross-section. The results indicate that the fusion zone has the least abrasive wear resistance, whereas the heat-affected zone (HAZ) exhibits the variable wear resistance. The resultant microstructure in the fusion zone is acicular ferrite with perlite and tempered martensite, and ferrite along with perlite in HAZ mainly brings the disparity in wear resistance.

The paper entitled “Processing of Haynes^®^ 282^®^ alloy by direct energy deposition with arc and wire” depicts the influence of arc energy input and shielding gas in the cold metal transfer (CMT)-based directed energy deposition process on mechanical and microstructural properties of Ni-based superalloy [7]. The arc energy is varied by changing wire feed speed and travel speed to produce thin wall and multi-track blocks. It is concluded that the risk of crack formation and lack of fusion is more pronounced for multi-track blocks than thin walls; however, it is significantly lower than Inconel 718. The samples produce higher strength and lower elongation at enhanced arc energy, whereas nitrogen-containing shielding gas produces higher hardness and low-impact energy and elongation.

The paper entitled “Optimization of CMT characteristic parameters for swing arc additive manufacturing of AZ91 magnesium alloy based on process stability analysis” illustrates the droplet transfer behavior of AZ91 magnesium alloy using swing arc formation by the CMT system [8]. The stability in metal transfer is identified by analyzing the electrical waveforms and droplet images. The horizontal component of arc force mainly influences the stable droplet transport. The process stability analysis of swing arc deposition is performed using the Vilarinho regularity index for short-circuit transfer metal transfer. It is concluded that the optimum combination of CMT parameters could provide a stable deposition process and an aesthetic appearance of the defect-free deposited layer.

The paper entitled “Measuring the cooling behavior of melt pools in (L-PBF) by pyrometry” shows the measurement of temperature using a pyrometer in a powder bed fusion process [9]. The additive manufacturing for a powder-based process is considered here for laser powder-based fusion (L-PBF). The instrument is calibrated by applying a thermal cycle to a printed track and measuring the temperature from both sides, i.e., from the pyrometer and thermocouple points. The comparison brings the calibrated single-color pyrometer data. A two-color pyrometer is also calibrated to enhance the precision of the results. Finally, the temperature is measured for an actual experiment on a single laser track where a distorted signal for smoke formation is considered. A correlation between cooling duration and process parameters such as laser power, scanning speed, laser focus size, and laser focus offset is established. It is concluded that the cooling rate or cooling duration from time–temperature simulation using the proposed pyrometer is helpful for understanding microstructural evolution.

The paper entitled “New partially water-soluble feedstocks for additive manufacturing of Ti6Al4V parts by material extrusion” describes the development of feedstock systems for Ti6Al4V from eco-friendly solvents such as water-soluble binder systems using fused filament fabrication (FFF) and fused feedstock deposition (FFD) processes [10]. The feasibility of two polar binder systems consisting of the low molecular weight polyethylene glycol and the high-molecular-weight polymers poly (vinylbutyral) or poly (methylmethacrylate) has been investigated. The rheological study indicates that a polar binder system can accommodate a solid load of 60 vol% of Ti6Al4V and reach 96% theoretical density after water debinding and thermal debinding and sintering of metal parts. It is concluded that further densification to 99% is possible to achieve after hot isostatic pressing.

It is evident from the presented papers that research on welding and additive manufacturing includes a wide range of manufacturing processes, materials, measurement, and characterization techniques. The fusion welding processes, such as laser welding, FCAW, MAG welding, gas-metal arc welding (GMAW), ultrasound-assisted fusion welding, and vacuum brazing, convey critical aspects and understanding to practical application. CMT is the most widely used metal transfer mechanism for the development of the wire arc additive manufacturing (WAAM) process. Heat transfer analysis, stress analysis, an analytical solution of jet force, electromagnetic force, ultrasonic vibration, and the application of optimization algorithms enrich the modeling approaches for welding and additive manufacturing processes. This Special Issue introduces the application of different materials, such as Ni-based superalloys, austenitic stainless steels, low-alloyed structural steel, martensitic steel, ferritic stainless steel, titanium alloy (Ti6Al4V), magnesium alloy, low- and high-molecular-weight polymers. A range of techniques like similar welding, dissimilar welding, multipass welding, droplet transport mechanism, and feedstock fabrication are learned from these papers. Several experimental evaluations like temperature measurement, temperature-dependent emissivity calibration, metallography and microstructure of metallic and non-metallic samples, images from high-speed cameras, mechanical properties, wear test of welded components, and recording of waveform current are essential outcomes from the presented papers. Till, there is much more new welding and joining technologies that are evolving day-by-day to meet the modern industry challenges. A much more demanding additive manufacturing technology for a wide variety of materials is also in the development stage, which may bring about a revolution in the industry.

## 3. Conclusions

The objective of this Special Issue effectively aims to improve the welding, joining, and additive manufacturing technologies; characterization of materials and processed components; and establishment of measurement techniques. It collectively shows how to produce beneficial microstructure and properties, avoid common defects, ensure the weldability and printability of commercial alloys, and address the need for post-processing to develop welding, joining, and additive manufacturing processes. The following conclusive statements are derived from the present editorial work. The vapor pressure in the laser welding process is the main driving force for keyhole formation. The microbubble formation through external vibration modifies the solidification behaviors in the fusion welding process. The inverse approach is the most suitable way to find the uncertain parameters of a computationally efficient heat source model. Vacuum brazing is an efficient method for joining laminated sheets of Inconel and stainless steel. A microstructurally sound dissimilar weld joint between austenitic and austenitic/ferritic steel can be achieved by fusion welding. The fusion zone of low-alloy martensitic steel produces the minimum wear resistance property. Heat input is a more effective method than nitrogen shielding gas to achieve high strength in Ni-based superalloy in the directed energy deposition process. A swing arc in the cold metal transfer process produces stable and defect-free deposition of magnesium alloy. A pyrometer-based temperature measurement technique is more flexible for additive manufacturing processes. The mechanism for the bonding of polymers in a non-conventional way and the joining of non-metallic materials shows the path to developing various printing technologies. However, rapid development of additive manufacturing demands much more research on the subject, which is currently the future of the manufacturing industry. In summary, the collection of articles in this Special Issue provides a foretaste view of the recent progress in specific areas of welding, joining, and additive manufacturing. This publication brings the opportunity for students, scientists, researchers, and entrepreneurs to exercise the subject and gain significant knowledge from it.
